# Serotonin and ATP4 are required for Wnt signaling and cilia-driven leftward flow in *Xenopus*

**DOI:** 10.1186/2046-2530-1-S1-P63

**Published:** 2012-11-16

**Authors:** M Blum, P Walentek, T Beyer, T Thumberger, M Tisler, B Ulmer, I Schneider, M Danilchik

**Affiliations:** 1 University of Hohenheim, Institute of Zoology, Stuttgart, Germany; 2 OHSU, Portland, USA

## 

In fish and mammalian neurula embryos midline epithelia (Kupffer's vesicle, posterior notochord/node) harbor polarized monocilia, which rotate in a clockwise manner to produce a leftward extracellular fluid flow. Flow induces asymmetric gene expression on the left side, resulting in asymmetric organ morphogenesis and placement. In frog, much earlier asymmetries were described for the monoamine serotonin and the ion pump gastric H+/K+-ATPase (ATP4). The 'ion-flux' model proposed that an ATP4-generated voltage gradient drives serotonin through gap junctions to asymmetrically enrich on one side of the embryo to break symmetry upstream of flow. Here we show that both serotonin and ATP4 were symmetrically expressed in *Xenopus* and required for Wnt-mediated setup of leftward flow. Flow and asymmetry were lost in embryos in which serotonin signaling was down-regulated. Serotonin was required as competence factor of Wnt signaling, which provides the instructive signal to specify the superficial mesoderm (SM), i.e. the structure which gives rise to the ciliated gastrocoel roof plate (GRP) where flow in frog occurs. Gene knock-down or pharmacological inhibition of ATP4 compromised organ situs, asymmetric gene expression and leftward flow. The GRP revealed fewer, shortened and misaligned cilia. FoxJ1, a master control gene of motile cilia, was down-regulated in the SM. Specifically, ATP4 was required for Wnt/ß-catenin regulated Foxj1 induction and Wnt/PCP dependent cilia polarization. Our work confirms flow as primary mechanism of symmetry breakage in frog and argues for evolutionary conservation of symmetry breakage in vertebrates.

